# Supplementary material for: Learning curve and surgical outcome of robotic assisted colorectal surgery with ERAS program
Source: Sci Rep. 2022 Nov 29;12:20566. doi: 10.1038/s41598-022-24665-w (PMC9709162; doi:10.1038/s41598-022-24665-w)
Supplement: Supplementary file 2 — Supplementary Information 2. [file 41598_2022_24665_MOESM2_ESM.zip › RAL Raw data-Ñ[▒K/132/HS_ñw╝╨░O▒Kñσ.pdf]

正式摘要(電子簽章：000000232412824)

W95 / W95 ☐英文標題 ☐【讀取】憑證

臺中榮民總醫院

出院病歷摘要

轉入號： 轉出號：

[redacted]

**(8)**流水編號	
**(9)**入院日期	110/12/10 (2021)
**(10)**轉科日期	
**(11)**出院日期	110/12/14 (2021) 住院天數計 4 日

診斷

- 入院：1. S-colon cancer, 30cm FAV
  2. HTN
  3. Arrhythmia, without medication control
- 出院：1. S-colon cancer s/p Robotic assist AR on 2021/12/11
  2. HTN
  3. Arrhythmia, without medication control

主訴

- Anemia for months

病史

- This is a 65 years old male patient with history of

  1. HTN

  2. Arrhythmia, without medication control

  According to the patient, he had regular health exam and noticed about anemia several months ago. He keep follow up his Hb level at our OPD, and then LGi scopy was done on 2021/11/29, which showed circumferential tumor over 30cm with lumen stenosis and easy tcough bleeding, and the biopsy reported adenocarcinoma.

  Under the impression of S-colon cancer, this time he was admitted to our ward for operation.

體檢發現

- Anal inspection:
  Recto digital examination:
  Sigmoidoscopy:
  Anoscopy:
  Abdomen:
  - General appearance :
    - easy looking , conscious clear , without cardiopulmonary distress
    - Vital signs自動帶入date 體溫(BT) 脈搏(P) 呼吸(R) 血壓(BP) 身高(BH) 體重(BW) BMI
      20211210 36.5℃ 79次/分 18次/分 131/62mmHg 172.9cm 81.5Kg 27.3
    - Vital signs: B.P. 131/62 mmHg , P.R. 79 /min , R.R. 18 /min , B.T. 36.5 ℃
  - HEENT :
    - Eyes: no anemic conjunctiva, no icteric sclera, normal EOM
    - ENT: no otorrhea, no rhinorrhea, no erythematous mucosa, no injected tonsil, no deformity
  - Neck: supple, freely movable, trachea midline,no deformity, no engorged jugular vein , no palpable thyroid or lymph node enlargement
  - Chest:
    - Inspection: Grossly normal, symmetric and fully expansion, no accessory muscle used, no operation scar, no spider angiomata, no gynecomastia
    - Palpation: no palpable mass or nodule, no subcutaneous emphysema, no tenderness
    - Ausculation: clear breathing sound

- Heart:
    - Palpation: no thrill, no heave
    - Percussion: normal heart size
    - Ausculation: regular heart beat, normal S1, S2, no gallop, no murmur
- Extremities: No deformity, full ranged of motion without limitation, no clubbing finger or nail cyanosis, no pitting edema, no ulcer or pigmentation, no obvious skin lesion, no varicose vein, no bone, joint or muscle tenderness

CRS CONDITION

- Karnofsky performance status: (100) Normal, no complaints, no evidence of disease
- PE of Abdomen:
- CEA: 1.04 ng/ml
- Imaging Studies:
- Colonoscope report: .
- Figures:
    1. colon & rectum

手術日期及方法

- 手術日期及方法20211211 1. Robotic (XI) assisted anterior resection
  2. Intraoperative colonoscopy : (-).
  3. High ligation (+) : At IMA root. Left colic artery and IMV were also ligated via Endoclips.
  4. Take down from the splenic flexure : (-)
  5. Robotic endoGIA (45-3.5) X2 + EEA (33mm) were used
  6. Proximal cut end: 10 cm, distal cut end: 5 cm
  7. No drainage tube was inserted
  8. ADHESION BARRIER FILM was placed below the central abdominal wound
- 手術結果及發現：
    - 1101211 1. Robotic (XI) assisted anterior resection 2. Intraoperative colonoscopy : (-). 3. High ligation (+) : At IMA root. Left colic artery and IMV were also ligated via Endoclips. 4. Take down from the splenic flexure : (-) 5. Robotic endoGIA (45-3.5) X2 + EEA (33mm) were used 6. Proximal cut end: 10 cm, distal cut end: 5 cm 7. No drainage tube was inserted 8. ADHESION BARRIER FILM was placed below the central abdominal wound 術前診斷:S COLON CA 術後診斷:Ditto.s/p robotic AR

      手術日期: 1101211 麻醉方式:

      手術名稱:1. Robotic (XI) assisted anterior resection 2. Intraoperative colonoscopy : (-). 3. High ligation (+) : At IMA root. Left colic artery and IMV were also ligated via Endoclips. 4. Take down from the splenic flexure : (-) 5. Robotic endoGIA (45-3.5) X2 + EEA (33mm) were used 6. Proximal cut end: 10 cm, distal cut end: 5 cm 7. No drainage tube was inserted 8. ADHESION BARRIER FILM was placed below the central abdominal wound

      術前診斷:S COLON CA

      術後診斷: Ditto.s/p robotic AR

住院治療經過

- After admission, he received Robotic assist AR on 2021/12/11. He could tolerate Bland II diet on 12/13. Under relative stable condition, he discharged on 12/14, OPD f/u.

合併症

一般檢查

- 累積報告：

DATE	TIME	ALB	TP	BIL.T	BIL.D	ALKP	AST	ALT	LDH	AMY	LIPASE	NH3	Ferritin	UA
1101210	1925			0.49				12						

DATE	TIME	WBC	RBC	HGB	HCT	MCV	PLT	BLST	NEUT	BAND	SEG	LYM	MON	EOS	BAS
1101210	1924	7810		12.2	38.0		286		73.7			14.7	7.0	4.0	0.6

DATE	TIME	NA	K	CL	BUN	CREAT	eGFR	CA	Mg	P	CRP	ESR	PCT	D-Dimer	LACTATE
1101210	1925	141	4.2		21	1.27	60.49	8.7							

DATE	TIME	GLUCOSE	HbA1C	CHOL	TG	HDL	LDL	KETONE	CK	CKMB	TRO-T	PT-P	APTT-P	NT-pro BNP
1101210	1923											10.6	27.0	

特殊檢查

病理報告

其它

出院時情況

- 15.計劃性出院

出院指示

藥名	劑量	劑量單位	用法	頻次	數量	數量單位	現況	天數	單位
(口)Takepron OD tab 30mg	1	TAB	PO	QDAC	7	TAB	TH	7	天
Ultracet tab	1	TAB	PO	Q6HPRN	28	TAB	TH	7	天

出院計劃

- 門診日期
  - date on 2021/12/21

主治醫師 2062F 蔣鋒帆 / 住院醫師 2339C 蔡濬樟

正式摘要(電子簽章：000000232412824)

W95 / W95

doctorRank:B
